# A Scheduling Scheme for Improving the Performance and Security of MU-MIMO Systems

**DOI:** 10.3390/s22145369

**Published:** 2022-07-19

**Authors:** Henry Carvajal, Nathaly Orozco, Stalin Cacuango, Paola Salazar, Edgar Rosero, Fernando Almeida

**Affiliations:** 1 Faculty of Engineering and Applied Sciences (FICA), Telecommunications Engineering, Universidad de Las Américas (UDLA), Quito 170124, Ecuador; henry.carvajal@udla.edu.ec (H.C.); stalin.cacuango@udla.edu.ec (S.C.); paola.salazar@udla.edu.ec (P.S.); edgar.rosero.jacome@udla.edu.ec (E.R.); 2 School of Electrical and Computer Engineering, University of Campinas (UNICAMP), Campinas 13083-970, Brazil; ferdaral@decom.fee.unicamp.br

**Keywords:** MU-MIMO, zero-forcing, scheduling, imperfect channel estimation, Rician fading

## Abstract

For the receiver of multiple-input multiple-output (MIMO) systems, linear detectors are an interesting option due to their good performance and low complexity. Nevertheless, MIMO systems lose diversity in exchange for eliminating interference when linear detectors are used. Aiming to maintain the system diversity while mitigating interference between users, this work proposes a scheduling scheme for the uplink of multiuser MIMO (MU-MIMO) systems that employ *A* antennas and the zero-forcing (ZF) detector at the receiver in the base station (BS). The channel model includes Rician fading and additive white Gaussian noise (AWGN) in an imperfect channel estimation scenario. The proposed scheme selects *U* users from a group of $U_{t}$ users to transmit simultaneously, so that the signal-to-noise ratio (SNR) is maximized. For this, an exact expression to evaluate the SNR of the users is obtained. With this result, the scheduling strategy is proposed. Results show that as $U_{t}$ increases, the outage probability (OP), and the bit error rate (BER) decrease as the system diversity increases, even when the system is completely loaded, i.e., when U=A. Moreover, it is shown that the scheduling scheme counteracts the imperfect channel estimation effects as $U_{t}$ increases. Finally, the proposed scheme is tested in presence of an external eavesdropper trying to decode the information sent by the users. The results show that the presented proposal allows for a reduction of the secrecy-outage-probability (SOP) as $U_{t}$ increases.

## 1. Introduction

Wireless communications networks have seen accelerated development since they have become essential for the communication of people and machines. As a result, new techniques are continually proposed for improving their performance in terms of reliability, security, and transmission rates. Among them, the multiple-input multiple-output (MIMO) technique has become a key technology for the development of new generation mobile networks [1,2,3].

MIMO involves the use of multiple antennas at the transmitter and at the receiver, or is implemented when multiple devices with one or multiple antennas are connected simultaneously, in the same radio resource, to a base station (BS) with multiple antennas. In the latter case, this technique is known as multiuser-MIMO (MU-MIMO). In particular, the antenna array at the receiver improves the system diversity, and the different transmission antennas increase the transmission rate. However, it is necessary to employ multiuser detectors at the receiver in order to mitigate interference between users.

There are several techniques that allow improving system diversity, however, there are some challenges related to its implementation. For instance, the mitigation of the channel estimation effects on the performance of these wireless systems is a topic of interest in the literature [4,5]. In [6], the bit error rate (BER) of systems with *N* receiving antennas (diversity branches) is evaluated in non-ideal channel estimation scenarios, where an estimator structure based on maximum-likelihood (ML) estimation is also presented. An expression to evaluate the BER is also obtained, which requires the evaluation of a single integral involving the moment-generating function (MGF) of the squared norm of the vector containing the channel gains. In [7], upper and lower bound symbol error rate (SER) expressions are derived for a wireless system employing two-dimensional constellations and diversity techniques in the presence of non-ideal channel estimation. Some works analyze the performance of MIMO systems that operate in scenarios with imperfect channel estimation. In [8], the authors derive expressions to evaluate the BER for the maximal-ratio combiner (MRC), the zero-forcing (ZF), and the minimum-mean-square-error (MMSE) detectors considering quadrature-amplitude-modulations (QAM) and different practical channel estimation strategies. As a consequence, errors appear in the wireless channel estimation. In [9], the impact of imperfect channel estimation in MIMO systems using relays is analyzed, and expressions to evaluate the outage probability (OP) are derived. In this work, the imperfect channel estimation effects are modeled as external interference/noise. In [10], the impact of imperfect channel estimation and antenna correlation on the performance of massive MIMO systems is evaluated. The authors show that errors in the channel estimation generate floors in the BER curves when they are plotted as a function of the signal-to-noise ratio (SNR), and that these floors cannot be eliminated as the SNR increases.

The performance of MIMO systems has typically been analyzed on Rayleigh fading channels [8,9,11], which emulates the random fading behavior (random signal attenuation) in scenarios with no line-of-sight (NLOS) between the transmitter and the receiver. However, in the literature, there are other statistical models that allow emulating the fading behavior in line-of-sight (LOS) scenarios. Among these models, the Rician distribution stands out [5,12]. It is imperative to indicate that the Rayleigh distribution is a particular case of the Rician distribution. In [13], the spectral efficiency of massive MIMO systems operating in Rician fading channels is evaluated through simulations. In this work, it is shown that reducing the service area and increasing the number of receiving antennas improves the system performance. For the same type of fading channels, in [14] it is shown that when the Rician *K* factor increases, the sum-rate capacity of the uplink of MIMO systems improves. It was also observed that when the number of antennas in the BS is increased, the undesirable effects of imperfect channel estimation are reduced. Further, in [15], the uplink of MIMO systems using MRC detectors in Rician fading channels is studied. Specifically, it was determined that as the Rician *K* factor increases, the adverse effects produced by imperfect channel estimation at the receiver are reduced. In [16], the performance of massive MIMO systems in correlated Rician fading is evaluated. The authors analyze spectral efficiency in systems composed of multiple cells considering various channel estimators. In [17], the performance of MIMO systems is analyzed considering different types of fading. Specifically, Rayleigh, Rice, Nakagami and Weibull statistical distributions are employed in this work for fading modeling. Through numerical simulations, the BER and the capacity of the system are analyzed in scenarios with and without perfect channel estimation.

Multiuser detectors play a fundamental role in the proper operation of MIMO systems. In the literature, different detectors have been proposed that are characterized by their trade-off between performance and implementation complexity. In [18], it is indicated that the use of linear detectors in MIMO receivers is an interesting alternative from the complexity point of view. This work focuses on the study of linear detectors for the uplink of MIMO systems operating in correlated Rician fading channels. In [19], a multicarrier MIMO system using linear detectors is evaluated in terms of the mean BER. Thus, approximate expressions are obtained to evaluate this performance indicator. The results show that the minimum-mean-square-error (MMSE) detector has a better performance than the zero-forcing (ZF) detector, but the MMSE implementation requires knowledge of the instantaneous noise power.

Several investigations aim to optimize the performance of MU-MIMO via scheduling algorithms, which select a group of users or assign radio resources to the users based on different criteria [20,21]. For instance, in [22], a channel allocation strategy for MU-MIMO systems using precoding techniques is proposed for broadcasting applications. In [23], a Rician fading channel is considered where the number of users is greater than the number of pilot sequences. In this context, a component identification algorithm is proposed. In [24], it is established that channel allocation algorithms are necessary for achieving good performance in MIMO and massive MIMO systems. In addition, a channel grouping and allocation algorithm based on LOS conditions is proposed. In [25], an algorithm for selecting users experiencing good channel conditions is presented, but users at the edges of cells are often ignored with this proposal. In [26], a scheduling scheme based on the individual gain of each user’s channel is proposed. Its performance is evaluated in terms of the OP. In all the aforementioned works, perfect channel estimation at the receivers is considered, which is not fulfilled in real scenarios [8]. Finally, in [27], the authors present a state-of-the-art analysis related to channel allocation (scheduling) algorithms for the downlink of massive MIMO systems. In particular, the authors present schemes based on capacity, Frobenius norm, conditional entropy, and volume, along with diagonalization pre-processing for eliminating interference between users. The performance of the schemes is analyzed in terms of the sum-rate capacity via simulations in presence of Rayleigh fading, and it is shown that capacity-based algorithms, such as the Frobenius norm algorithm, guarantee the best sum-rate for the MIMO system.

A critical aspect that has gained attention in new generation mobile networks is information security, mainly for devices with low processing capacity, since here it is not possible to use complex encryption algorithms [28]. Therefore, physical layer security (PLS) becomes an interesting alternative for providing security in these scenarios [29]. In particular, PLS proposals must be adapted to work in conjunction with MIMO, because the latter technique must be implemented in a mandatory way to ensure the key-performance-indicators (KPIs) established for fifth-generation (5G) and beyond 5G (B5G) networks [30]. As a consequence, PLS in MIMO systems has recently been investigated in the literature. In [31], PLS is studied in MIMO systems that employ singular value decomposition (SVD) schemes, where it is determined that the higher the distance between an eavesdropper and the transmitter/receiver, the higher the system secrecy rate. In [32], the secrecy outage probability (SOP) of diversity schemes that employ antenna-user selection and user selection with space-time block coding (US-STBC) over Nakagami fading channels is analyzed. A similar scenario is investigated in [33], where the impact of the number of users, eavesdroppers, and antennas on secrecy performance is demonstrated by employing an asymptotic analysis. In both works, the single-user MRC technique is considered. Finally, in [34], transmit antenna selection is also considered in the downlink of a MIMO system in the presence of some trusted and untrusted users. In the system model, the BS selects a desired legitimate user and the transmitting antenna pair. Then, once the instantaneous SNR of the previously selected legitimate user is below a threshold, the legitimate user with the highest SNR is selected for transmitting in the next symbol period. In all these works, scenarios with perfect channel estimation at the receiver are considered.

Table 1 summarizes the MIMO research areas on which the contributions of previous cited works are focused. Based on this literature review and to the best of the authors’ knowledge, imperfect channel estimation has not been previously considered when channel scheduling schemes have been proposed or analyzed in MIMO systems operating over generalized fading channels, such as Rician fading. In addition, scheduling schemes constitute an interesting technique for guaranteeing security in the physical layer of MIMO systems. However, these schemes have been little explored so far in the literature for this purpose. Therefore, this is the main motivation for the proposal presented in this work.

By the above, this paper proposes a novel scheduling scheme for the uplink of MU-MIMO systems using the multiuser ZF detector at the receiver in the BS considering the presence of imperfect channel estimation at the receiver. At this point, it is important to indicate that it is well known that the ZF detector loses diversity in exchange for eliminating interference [35], and therefore, the proposed scheme aims to maintain system diversity while mitigating interference between users. In addition, it seeks to counteract the undesirable effects produced by the imperfect channel estimation. The presented proposal is evaluated in a Rician fading channel in terms of the mean BER and the OP. In addition, the computational complexity of the presented scheduling scheme is analyzed. Finally, the proposed scheme is employed in a network with the presence of an external eavesdropper trying to decode the information sent by the users. In this case, the system performance is analyzed in terms of the SOP.

The remainder of this work is organized as follows. The system model is detailed in Section 2. The instantaneous SNR for each user is obtained in Section 3. The proposed scheduling scheme is presented in Section 4, where its computational complexity is also analyzed. In addition, this section introduces some aspects related to other proposals from the literature. Then, Section 5 presents representative numerical results and discussions, where the performance of the proposed scheduling algorithm is also compared with the performance of other proposals from previous works. Finally, the main conclusions of this work are summarized in Section 6.

In the following *x*, x and X represent scalar, vector and matrix, respectively. Further, ${\left(\cdot \right)}^{†}$ denotes pseudo-inverse, ${\left(\cdot \right)}^{-1}$ denotes matrix inversion, ${\left(\cdot \right)}^{H}$ represents conjugate-transpose, E[·] is the expectation operator, Var[·] is the variance operator, the function max[x] obtains the maximum value from x, ⌈·⌉ denotes ceil operation, $f_{x}\left(x\right)$ is the probability density function (PDF) of the random variable *x*, ${∥x∥}_{F}$ is the Frobenius norm of x, ${\left[X\right]}_{k,\ell }$ indicates the element in the *k*-th row and *ℓ*-th column of X, and $j=\sqrt{-1}$ is the imaginary unit.

## 2. System Model

This section describes the system model, which includes the channel model and the structure of the received signals, the ZF detector description, and the imperfect channel estimation criterion used.

### 2.1. Channel Model and Received Signals

Consider the uplink of a single-cell MU-MIMO system that employs *A* antennas at the BS to serve *U* user terminals (UTs), each of them equipped with a single antenna, such that A≥U. The *U* UTs transmit simultaneously over the same frequency channel (subcarrier), which has a bandwidth less than the channel coherence bandwidth, $B_{c}$. Therefore, the transmitted signals are affected by flat fading (This scenario appears in multicarrier systems that employ orthogonal-frequency-division-multiplexing (OFDM), where the total bandwidth used by the system is greater than $B_{c}$, but the bandwidth of each subcarrier is much less than $B_{c}$. This, along with the use of a cyclic prefix allows removal of inter-symbol interference (ISI) and inter-carrier interference (ICI), and guarantees that each subcarrier is affected by flat fading [36].) [5]. Under these premises, the vector of samples received in the BS during a symbol interval y of dimensions A×1, can be written as

(1) $$\begin{array}{cc} y & =\mathrm{Hs}+n=\sum_{k=1}^{U}h_{k}s_{k}+n, \end{array}$$

where s is a U×1 vector containing the transmitted symbols. Thus, $s_{k}$ is the symbol transmitted by the *k*-th user and $H=[h_{1}\;h_{2}\;\cdot \cdot \cdot \;h_{U}]$ is an A×U matrix containing the wireless channel gains. Therefore, the column vector $h_{k}$ contains *A* channel gains affecting the *k*-th user transmission. The elements of $h_{k}$ are complex Gaussian random variables, i.e., $h_{a,k}=g_{1}+jg_{2}$, such that $g_{1}$ and $g_{2}$ are real Gaussian random variables with mean $\mu /\sqrt{2}$ and variance $\sigma^{2}$. Then, the channel gains can be rewritten as $h_{a,k}=\alpha_{a,k}\exp\left(j\phi_{a,k}\right)$, for a=1,2,...,A, and k=1,2,...,U, where $\alpha_{a,k}$ is the fading amplitude modeled by a Rician random variable, whose PDF is defined by [37]

(2) $$f_{\alpha }\left(\alpha \right)=\frac{2(K+1)}{P}\alpha \exp\left(-\frac{K+1}{P}\alpha^{2}-K\right)I_{0}\left(2\sqrt{\frac{K(K+1)}{P}}\alpha \right),\;\alpha \geq 0,$$

where $I_{0}\left(\cdot \right)$ is the modified Bessel function of the first type and order zero [38] (Equation (9.1.10)), and 

(3) $$\begin{array}{c} K=\frac{\mu^{2}}{2\sigma^{2}}, \end{array}$$

is a shape parameter and represents the ratio of the power of the LOS path and the power of the remaining non-LOS multi-path. Further, the scale parameter

(4) $$\begin{array}{c} P=\mu^{2}+2\sigma^{2}, \end{array}$$

indicates the power received on all paths. In addition, $\phi_{a,k}$ is the random channel phase. By employing the joint distribution of α and ϕ [37] (Equation (6-74)), the marginal PDF of ϕ can be obtained as

(5) fΦ(ϕ)=∫0∞f(α,ϕ)dα=12exp−K1+K1+πexpK1+Kcos2(θ)erfK1+K×erfc−K1+Kcos(θ)K1+Kcos(θ),

where erfc(·) is the complementary error function [38] (Equation (7.1.2)).

Finally, in (1), n is the additive white Gaussian noise (AWGN) vector, whose elements are independent and identically distributed complex Gaussian random variables with zero mean and variance $\sigma_{n}^{2}$, that is, $\mathrm{CN}(0,\sigma_{n}^{2})$, where $\sigma_{n}^{2}=N_{0}/\left(2T_{s}\right)$ is the noise variance, $N_{0}$ is the unilateral noise power spectral density, and $T_{s}$ is the symbol duration.

### 2.2. Multi-User Detector

The receiver at the BS employs the ZF multiuser detector that performs a linear combination of the received samples in y using a matrix W of dimensions U×A, such that interference between users is completely eliminated, even though this may increase the noise variance. Thus, the ZF detector chooses W so that WH=I, where I is the identity matrix.

Note that W exists when H is not range-deficient, that is, when A≥U. Further, W exists when the columns of H are linearly independent. This condition is fulfilled in the system model as long as the separation of the antennas in the BS and the random location of the users in the cell guarantees independent channel gains. (Theoretically, an antenna spacing of λ/2 guarantees independent channel gains, where λ is the signal wavelength [5]). When A=U, H is a square matrix, and in that case the solution of the ZF criterion is unique and is $W=H^{-1}$. On the other hand, when A>U, there is an infinite number of matrices W that satisfy that WH=I. In these cases, the ZF detector uses a matrix W such that WH=I, which also minimizes the root mean square error, that is, $E[∥\mathrm{Wy}-s{∥}^{2}]$ [36].

Since H presents sufficient statistics [4], W can be decomposed into the product $W={\mathrm{XH}}^{H}$, where X is a dimensional matrix U×U to be determined next. Thus, the restriction WH=I becomes ${\mathrm{XH}}^{H}H=I$. Since $H^{H}H$ is invertible, it can then be determined that $X={\left(H^{H}H\right)}^{-1}$. Therefore,

(6) $$W=H^{†}={\left(H^{H}H\right)}^{-1}H^{H},$$

which is the Moore–Penrose pseudo-inverse matrix of H. If H is square, then W is reduced to $H^{-1}$.

In the BS receiver, the linear transformation can be implemented via a filterbank [39]. For the presented scenario, the output of the ZF detector before the demapper (a structure used in the receiver to estimate the transmitted symbol based on the minimum distance criterion [36]) is obtained by applying

(7) $$\begin{array}{c} z=\mathrm{Wy}. \end{array}$$

### 2.3. Imperfect Channel Estimation

The ZF detector requires knowledge of H, defined in (1), to recover the transmitted symbol vector. In practice, channel gains are estimated by transmitting pilot symbols that are known at the receiver [40]. However, the pilots can be affected by noise, delay, or synchronization problems. Therefore, it is necessary to employ channel estimation techniques in practical systems [41]. One of the most used estimation techniques due to the trade-off between complexity and performance is based on the MMSE criterion [40], that is based on linear operators that minimize the mean squared error (MSE) between the real channel gains and the estimated ones. In particular, the MMSE estimator is an adequate estimator for jointly Gaussian distributed random variables. However, it is often used in non-Gaussian scenarios and performs well for different distributions that are not too different from a Gaussian distribution [42]. In this sense, and considering that the channel gains, i.e., the entries of H, can be modeled as Gaussian random variables, some works have considered this estimator for MIMO systems [41,42,43,44,45]. In practice, the MMSE estimation is not perfect and, therefore, there is residual interference within the process. In this case, ref. [43] (Equation (2)) shows that the estimated channel matrix can be written as

(8) $$\hat{H}=\sqrt{1-e^{2}}\;H+e\Omega ,$$

where Ω is an A×U matrix whose elements are complex Gaussian random variables, independent and identically distributed with zero mean and unitary variance [43]. Furthermore, the elements of Ω are independent of the elements of H. Thus, the parameter *e*, which assumes values in the interval [0,1], represents the level of error in the channel estimation process. Therefore, e=0 implies perfect channel estimation and e=1 implies that there is a complete error in the channel estimation.

By the above, from (7) and (8), the vector of samples at the output of the ZF detector can be written as

(9) $$z={\hat{H}}^{†}y=s+{\hat{H}}^{†}n.$$

## 3. Instantaneous Signal-to-Noise Ratio

From (9), the variance of the *k*-th element of z, that is, $z_{k}$, conditioned on the instantaneous elements of $\hat{H}$, can be found as follows

(10) $$\begin{array}{cc} Var[z_{k}|\hat{H}] & =E{\left[\left({\hat{H}}^{†}+n\right){\left({\hat{H}}^{†}+n\right)}^{H}|\hat{H}\right]}_{k,k}\\ \; & ={\left[{\hat{H}}^{†}E\left[nn^{H}\right]{\left({\hat{H}}^{†}\right)}^{H}\right]}_{k,k}\\ \; & =\sigma_{n}^{2}{\left[{\hat{H}}^{†}{\left({\hat{H}}^{†}\right)}^{H}\right]}_{k,k}\\ \; & =\sigma_{n}^{2}{\left[{\left({\hat{H}}^{H}\hat{H}\right)}^{-1}\right]}_{k,k}, \end{array}$$

where the usage is that ${\left(\mathrm{AB}\right)}^{H}=B^{H}A^{H}$, that $E\left[nn^{H}\right]=\sigma_{n}^{2}I_{A}$, and where $I_{A}$ is a A×A identity matrix. Finally, in the last step, the identity ${\hat{H}}^{†}{\left({\hat{H}}^{†}\right)}^{H}={\left({\hat{H}}^{H}\hat{H}\right)}^{-1}$ is employed [46].

From (10), the instantaneous SNR at the output of the ZF detector for the *k*-th user can be written as

(11) $$\begin{array}{c} \gamma_{k}=\frac{s_{k}^{2}}{2Var[z_{k}|\hat{H}]}=\frac{s_{k}^{2}}{2\sigma_{n}^{2}}\zeta_{k}=\frac{E_{b}}{N_{0}}\zeta_{k}\log_{2}M, \end{array}$$

where $\zeta_{k}$ is a random variable that can be written as

(12) $$\begin{array}{c} \zeta_{k}=\frac{1}{{\left[V^{-1}\right]}_{k,k}}, \end{array}$$

where the U×U matrix V is obtained as $V={\hat{H}}^{H}\hat{H}$. In addition, it was considered that the energy received per symbol is $E_{s}=s_{k}^{2}T_{s}/2$ and that the energy received per bit is equal to $E_{b}=E_{s}/\log_{2}\left(M\right)$, where *M* is the modulation order. Consequently, $\log_{2}M$ is the number of bits transmitted in each symbol.

## 4. Scheduling Schemes

In this section, two existing suboptimal scheduling schemes are first introduced for comparison purposes. Then, a novel a scheduling scheme is proposed in order to optimize the performance of MU-MIMO systems that employ the ZF detector.

### 4.1. Suboptimal Schemes from Previous Works

As indicated in Section 1, some scheduling schemes for MIMO systems have been proposed in the literature. Below, two suboptimal schemes are detailed, which have a good trade-off between performance and complexity, in order to make a comparison in terms of performance with the scheme proposed in this work.

#### 4.1.1. Frobenius Norm-Based Scheduling Algorithm

This algorithm was initially proposed in [20] and is analyzed in detail in [47]. Based on the latest work, in which it is assumed that each user terminal has $A_{u}$ antennas, and considering that the BS has *A* receiving antennas, the algorithm first selects the user with the maximum channel energy, that is,

(13) $$u_{0}=\underset{k}{\arg\max}{∥H_{k}∥}_{F}^{2},$$

for $k\in \{1,2,\ldots ,U_{t}\}$, where $U_{t}$ is the total number of users competing to transmit, such that $U_{t}\geq U$ and $H_{k}$ is an $A\times A_{u}$ matrix containing the channel gains of the *k*-th user (To keep concordance with the proposed system model, $A_{u}=1$ is employed). Then, the algorithm selects the next user that provides the maximum sum of the equivalent channel energy together with the selected users. This procedure can be represented as [47]

(14) $$u_{i}=\underset{k\in \;\Psi }{\arg\max}\sum_{\ell \in \{k\}\cup \Upsilon }{∥H_{\ell }^{\mathrm{eq}}∥}_{F}^{2},$$

where Υ and Ψ represent the selected and non-selected user groups, respectively. Further, $H_{\ell }^{\mathrm{eq}}$ is an equivalent channel matrix generated from the product of the original channel matrix and a precoding matrix [47] (Equation (6)). Finally, $u_{i}$ denotes the user selected during the *i*-th interaction. The algorithm ends when *U* users are selected.

Considering that $A_{u}=1$, the total number of complex operations (i.e., complex additions and complex multiplications) performed by this algorithm is [20] (Equation (7))

(15) $$\begin{array}{cc} O_{f}= & \sum_{\ell =2}^{\left\lceil U/A\right\rceil }\left\{\left[8{\left(\ell -1\right)}^{3}+18{\left(\ell -1\right)}^{2}+18\left(\ell -1\right)\right]A^{2}U\right.\\ \; & \left.+[2{\left(\ell -1\right)}^{2}+4\left(\ell -1\right)]AU\right\}\left(U_{t}-\ell +1\right)+4U_{t}AU. \end{array}$$

#### 4.1.2. “Fair” Channel Allocation Algorithm

This scheme was proposed in [26] and, similar to the algorithm based on Frobenius norms, the system chooses, in a first transmission (first time slot), the *U* users whose channel gain vectors have the highest squared Frobenius norms. Then, in the subsequent transmission, the algorithm chooses the *U* users whose channel gain vectors have the smallest squared Frobenius norms. Subsequently, the process is repeated. Alternate selection of users with higher and lower channel gains ensures fair access to the radio resources among all active users.

This algorithm is characterized by its low computational complexity, since only the Frobenius norms of the vectors that contain the channel gains for the $U_{t}$ users must be calculated. Thus, calculating the squared Frobenius norm of a vector of dimensions A×1 requires 2A complex additions and 2A complex multiplications. Therefore, the total number of complex operations required by this algorithm is equal to

(16) $$O_{j}=4AU_{t}.$$

### 4.2. Proposed Scheduling Scheme

**Figure 1:**
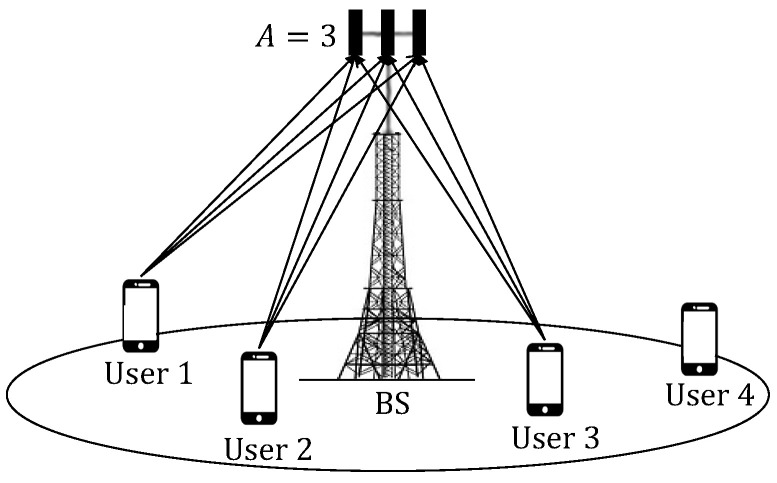
MU-MIMO system with A=3 antennas in the BS, in which U=3 users are served simultaneously out of a total of $U_{t}=4$ users.

A MU-MIMO system that employs the ZF detector at the receiver can serve a number of users less than or equal to the number of receiving antennas, as shown in Figure 1. With the ZF detector, the system diversity is D=A−U+1 [8,35]. In particular, the greater the diversity, the better the system performance. Thus, if the number of users is equal to the number of antennas in the receiver, the detector eliminates the interference but loses the spatial diversity, namely, the performance for all users is similar to the performance of a system in which there is only one transmitting and one receiving antenna [8].

Now, consider a scenario where the total number of users $U_{t}$ is greater than the number of antennas *A*, as in the example shown in Figure 1, where A=3, $U_{t}=4$ and U=3. In this case, the system can select a group of *U* users who will transmit simultaneously over the same radio resource. Thus, a scheduling scheme is proposed below to select *U* users among the $U_{t}$ users competing to transmit. In addition, this channel allocation scheme along with the ZF detector is intended to eliminate interference, but also to guarantee some diversity in order to improve system performance.

**Figure 2:**
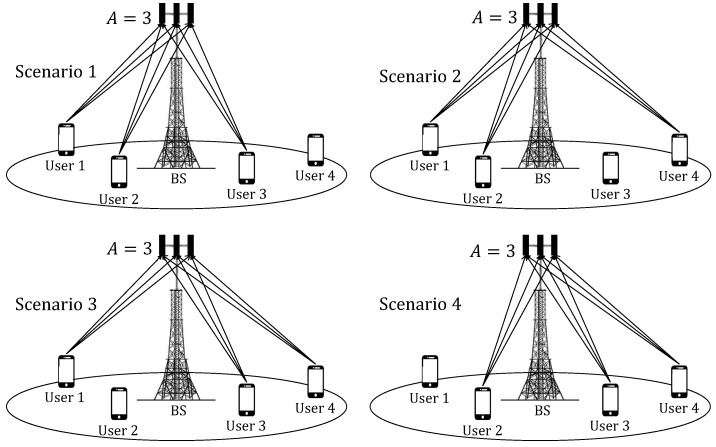
Channel assignment scenarios with the proposed scheduling scheme considering $U_{t}=4$ and U=3 for A=3 receiving antennas.

Consider as an example Figure 2, where four different user selection scenarios appear. The proposed scheme considers the wireless communication channel for all users at once, that is, it employs the complete estimated channel matrix $\hat{H}$ to decide which users should transmit, and consequently, considers the instantaneous SNR of each user, which was obtained in (11). This approach differs from other works in which the channel gains of each user are considered separately [26], and not the whole matrix $\hat{H}$ altogether.

Let U be a matrix in which each column is one of the combinations of *U* different integer numbers taken from the subset $I=\{1,2,\ldots ,U_{t}\}$. Therefore, U has *U* rows and UtU columns, where xy=x!(x−y)!y!. Moreover, let $u_{\ell }$ be the *ℓ*-th column of U. Associating each of the $U_{t}$ candidate users with each integer in the subset I, ${\hat{H}}_{u_{\ell }}$ can be defined as a matrix similar to $\hat{H}$ but containing only the channel gains for the users defined by the vector $u_{\ell }$. For a better understanding, consider again as an example the scenario with $A=3,U_{t}=4$ and U=3. The matrices U and ${\hat{H}}_{u_{2}}$, respectively, are given by

(17) $$U=\left(\begin{array}{cccc} 1 & 1 & 1 & 2\\ 2 & 2 & 3 & 3\\ 3 & 4 & 4 & 4 \end{array}\right),\;{\hat{H}}_{u_{2}}=\left(\begin{array}{ccc} {\hat{h}}_{1,1} & {\hat{h}}_{1,2} & {\hat{h}}_{1,4}\\ {\hat{h}}_{2,1} & {\hat{h}}_{2,2} & {\hat{h}}_{2,4}\\ {\hat{h}}_{3,1} & {\hat{h}}_{3,2} & {\hat{h}}_{3,4} \end{array}\right),$$

where ${\hat{h}}_{a,k}$ is the estimated channel gain at the receiver for the *a*-th antenna and the *k*-th user.

The SNR for the *k*-th user is proportional to $\zeta_{k}$, defined in (12). Therefore, every possible matrix ${\hat{H}}_{u_{\ell }}$ must be taken for ℓ=1,2,…UtU, and the related random variables $\zeta_{k}$ must be determined. Then, the scenario in which the instantaneous SNR of the *U* users is maximized is the one in which the sum of the variables $\zeta_{k}$ is the greatest, assuming that the $E_{b}/N_{0}$ ratio is the same for all users. (This assumption is valid, since current cellular systems perform almost perfect power control on the uplink [48] and therefore, signals from different users arrive with similar power at the BS.) By the above, the following metric allows us to determine the sum of the random variables $\zeta_{k}$ for each matrix ${\hat{H}}_{u_{\ell }}$,

(18) $$\begin{array}{c} M\left(u_{\ell }\right)=\sum_{k=1}^{U}\frac{1}{{\left[{\left({\hat{H}}_{u_{\ell }}^{H}{\hat{H}}_{u_{\ell }}\right)}^{-1}\right]}_{k,k}},\;for\;\ell =1,2,...,\left(\begin{array}{c} U_{t}\\ U \end{array}\right). \end{array}$$

Then, the MU-MIMO system selects the set of users based on the following criteria

(19) $$\tilde{u}=\underset{u_{\ell }}{\arg\max}\;M\left(u_{\ell }\right).$$

As result, $\tilde{u}$ contains the indices of the *U* users that must transmit. It is important to notice that the order of the columns for different matrices ${\hat{H}}_{u}\ell$ does not modify the final decision made by the proposed scheme. For this reason, the number of options evaluated by the scheduler is specifically limited to UtU.

Algorithm 1 summarizes the process for finding vector $\tilde{u}$ as a pseudocode.
**Algorithm 1** Scheduling algorithm pseudocode**Input:** 
$U,U_{t},\hat{H}$  1:Create matrix U with dimensions U×UtU (see as reference eq. (17))  2:**for**ℓ=1,2,…,UtU**do**  3:   Take the *ℓ*-th column from U, i.e, $u_{\ell }$  4:   Take the columns of $\hat{H}$ based on the elements of $u_{\ell }$ and create matrix ${\hat{H}}_{u_{\ell }}$  5:   acum = 0  6:   **for** k=1,2,…,U **do**  7:     acum = acum + ${\left\{{\left[{\left({\hat{H}}_{u_{\ell }}^{H}{\hat{H}}_{u_{\ell }}\right)}^{-1}\right]}_{k,k}\right\}}^{-1}$  8:   **end for**  9:   $M\left(u_{\ell }\right)=$ acum 10:**end for** 11:Select $u_{\ell }$ that generates $max\left\{M\left(u_{\ell }\right)\right\}$ for ℓ∈1,2,…,UtU 12:$\tilde{u}=u_{\ell }$**Output:** 
$\tilde{u}$ =0

Notice that (18) and (19) allows selecting the *U* users with the highest SNR after the multiuser detection process, that is, the *U* users with the highest SNR at the ZF detector output. (Selecting the *U* users, among the $U_{t}$ users, whose sum of SNRs is maximum, is equivalent to selecting the *U* users with the highest individual SNRs.) From the literature, it is known that a higher SNR implies a higher channel capacity or a lower BER. In this sense, the presented proposal allows the guarantee of better performance for the users in the MIMO system. In addition, the proposed scheduling scheme differs from others in the literature in which the SNR is considered at the receiver input and, therefore, only the channel gains are considered before the processing performed by the multiuser detector, as evidenced in (13) and (14).

When the MIMO system is not fully loaded, that is, when A>U, the ZF multiuser detector alone can guarantee a certain diversity order for the MU-MIMO system. In this case, the proposed allocation scheme will further increase the system diversity. However, the presented proposal can be more attractive if A=U, since it improves the system diversity even when the system is fully loaded. From the literature, it is known that when system diversity increases, then performance improves, which implies that lower SNR values or higher modulation orders can be used in the system and still guarantee an adequate performance [36]. These aspects are validated in Section 5.

Finally, it could be thought that one or more UTs do not transmit with the proposed scheme. This can be interpreted as unfair to UTs whose channel gains are weaker. At this point, it is important to notice that, according to 3GPP Release 15, mobile systems under the 5G standard employ the OFDM technique in the uplink [49]. Therefore, if it is considered that the proposed scheme operates with that standard, there are several subcarriers available for users to transmit. In addition, the total system bandwidth is typically greater than $B_{c}$, thus, subcarriers separated by a frequency interval greater than $B_{c}$ will be affected by channel gains (or equivalently flat fading) that are independent [50]. Consequently, the channel gain for a user may be weaker in some subcarriers, but stronger in others. Therefore, a UT that does not transmit on a subcarrier can transmit on another, which guarantees that all users within the same cell can transmit.

### 4.3. Computational Complexity Analysis of the Proposed Scheduling Scheme

Now, the computational complexity of the proposed scheduling scheme is analyzed based on the number of complex operations performed. For this, let A, B, and C be arbitrary complex matrices with dimensions m×n, n×p and m×m, respectively. Thus, the product AB has mp(n−1) complex additions and mnp complex multiplications. In addition, the inversion of C requires $\frac{1}{3}m^{3}+\frac{1}{2}m^{2}-\frac{5}{6}m$ complex additions and subtractions and $\frac{1}{3}m^{3}+m^{2}-\frac{1}{3}m$ complex multiplications and divisions considering that Gaussian elimination with reverse substitution is used [51]. In the literature, there are several techniques to reduce the complexity of matrix operations, however, in the computational complexity analysis carried out in this work these techniques have not been considered.

With the above result, and knowing that $\hat{H}$ has dimensions A×U and that the vector y has dimensions A×1, by considering (9), the number of complex additions and subtractions required by the ZF detector to calculate the product ${\hat{H}}^{†}y={\left({\hat{H}}^{H}\hat{H}\right)}^{-1}{\hat{H}}^{H}y$, when A>U, is $\frac{1}{3}U^{3}+\left(2A-\frac{1}{2}\right)U^{2}-\frac{11}{6}U$. In addition, the number of complex multiplications and divisions required to perform such an operation is $\frac{1}{3}U^{3}+\left(2A+1\right)U^{2}+\left(A-\frac{1}{3}\right)U$. On the other hand, if $\hat{H}$ is a square matrix, the ZF detector performs the operation ${\hat{H}}^{-1}y$. In this case, the detector performs $\frac{1}{3}U^{3}+\frac{3}{2}U^{2}-\frac{11}{6}U$ complex additions and subtractions and $\frac{1}{3}U^{3}+2U^{2}-\frac{1}{3}U$ complex multiplications and divisions.

Next, the computational complexity of the scheduling criterion defined in (18) and (19) is analyzed. The number of complex additions and subtractions needed to calculate ${\hat{H}}_{u}\ell^{H}{\hat{H}}_{u}\ell$ and its inverse are $\left(A-1\right)U^{2}$ and $\frac{1}{3}U^{3}+\frac{1}{2}U^{2}-\frac{5}{6}U$, respectively, and the number of complex multiplications and divisions required are $AU^{2}$ and $\frac{1}{3}U^{3}+U^{2}-\frac{1}{3}U$, respectively. Also, notice that (18) requires *U* divisions and *U* sums for each *ℓ*. Therefore, (19) requires $\left(\begin{array}{c} U_{t}\\ U \end{array}\right)$$\left[\frac{1}{3}U^{3}+\left(A-\frac{1}{2}\right)U^{2}+\frac{1}{6}U\right]$ complex additions and subtractions and $\left(\begin{array}{c} U_{t}\\ U \end{array}\right)$$\left[\frac{1}{3}U^{3}+\left(A+1\right)U^{2}+\frac{2}{3}U\right]$ complex multiplications and divisions. Thus, the total number of complex operations required by the proposed algorithm is

(20) $$O_{p}=\left(\begin{array}{c} U_{t}\\ U \end{array}\right)\left[\frac{2}{3}U^{3}+\left(2A+\frac{1}{2}\right)U^{2}+\frac{5}{6}U\right].$$

In the above expression, notice that the binomial coefficient UtU determines the number of operations performed by the algorithm. It can be shown that the maximum binomial coefficient value is obtained when $U=U_{t}/2$ for $U_{t}$ even and $U=(U_{t}-1)/2$ or $U=(U_{t}+1)/2$ for $U_{t}$ odd. As a consequence, the computational complexity of the proposed algorithm can be high in these scenarios. Therefore, the system must be able to select an appropriate value of $U_{t}$ based on the number of users that can be served in each radio resource, that is, *U*, and based on the computational capacity of the receiver. In addition, although a mobile network can serve a large number of users in each cell, this does not necessarily imply that all those users are competing to transmit on the same radio resource, since each cell has several radio resources available. Therefore, this allows the value of $U_{t}$ to be a parameter selected directly by the BS based on its computational capacity characteristics and based on the system operation parameters. Moreover, it is important to remember that the presented algorithm is an interesting proposal in scenarios in which the system is fully loaded. Thus, its use is expected to be performed in scenarios where the difference between $U_{t}$ and *U* is not high and, therefore, the number of operations carried out by the scheduling algorithm can be executed in practical systems. Despite the small difference between $U_{t}$ and *U*, the MIMO system performance can be improved considerably, as discussed in the next section.

## 5. Numerical Results and Discussions

In this section, the performance of the proposed scheme is analyzed using numerical results obtained through Monte-Carlo simulations with $5\times 10^{7}$ trials in some representative scenarios that consider different operating parameters (The base algorithms in Matlab^®^ employed to generate the numerical results are available at this link https://drive.google.com/drive/folders/12qzMgK5Oq5PSbTMAnSvPYkXLRM-3Tbi6?usp=sharing (accessed on 11 July 2022)).

For simulation purposes, a MIMO system with *A* antennas at the BS and serving *U* users is assumed, where the transmitted symbols belong to a constellation with normalized mean power, i.e, $E[s_{k}^{2}]=1$. In addition, it is considered that all users transmit simultaneously over the same frequency subcarrier from a wireless system operating over flat fading, i.e., there is no presence of ICI or ISI (See Section 2.1). For the Rician fading, it is assumed that $2\sigma^{2}=1$, which from (3) implies that the mean power of the non-line-of-sight multipath has been normalized. The performance is analyzed in terms of the OP, the BER and the SOP of the MIMO system, in the last case considering the presence of a spy receiver (eavesdropper). More details related to the simulation parameters are presented in the description of each figure.

### 5.1. Outage Probability

**Figure 3:**
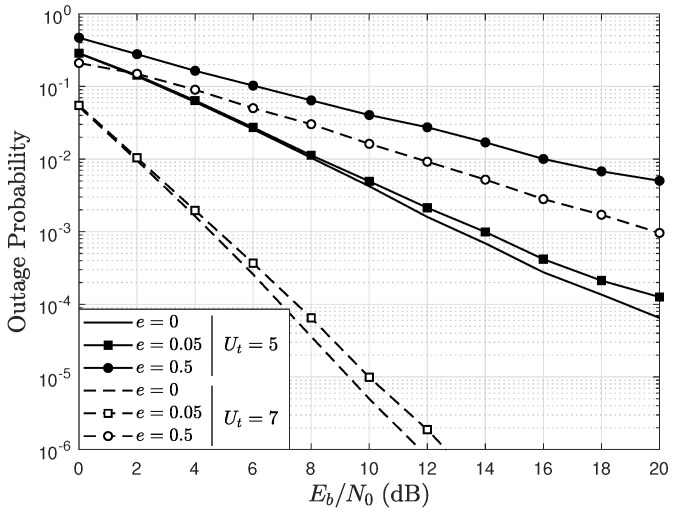
OP as a function of $E_{b}/N_{0}$, parameterized by $U_{t}$ and by *e*, considering BPSK modulation, A=4, U=4, T=0 dB, and a channel with Rician factor K=1.

The OP is defined as the probability that the instantaneous SNR of each user is below a given threshold *T*. Thus, Figure 3 shows the OP as a function of $E_{b}/N_{0}$, parameterized by $U_{t}$, and by *e* considering K=1, U=4, A=4 antennas, binary phase shift keying (BPSK) modulation (M=2) and a threshold T=0 dB. In the figure, it can be observed that as $E_{b}/N_{0}$ increases, the OP decreases. Moreover, when $U_{t}$ increases, the system performance improves, since there are more users competing to transmit and, therefore, the proposed scheduling scheme can find a better combination of UTs that maximizes the SNR, which translates into a lower OP. For a given value of $U_{t}$, notice that when *e* increases, the OP also increases. This is because the error in the channel estimation increases. However, for a given value of *e*, the system performance improves when $U_{t}$ increases. For instance, for e=0.05 and $E_{b}/N_{0}=15$ dB, the OP with $U_{t}=5$ is equal to $2.05\times 10^{-3}$, and with $U_{t}=7$, it decreases to $1.98\times 10^{-6}$. For e=0.5, $E_{b}/N_{0}=20$ dB and $U_{t}=5$, the OP is equal to $4.8\times 10^{-3}$, and when $U_{t}=7$ the OP is reduced to $9.3\times 10^{-4}$. However, the performance improvement is greater for small values of *e*, since there is a better channel estimation and the diversity that the proposed scheduling scheme guarantees can be better employed for the MIMO system. Specifically, high values of *e* may cause some users to not be properly selected by the algorithm, which has a direct impact on the performance gain. Even so, it is observed in the results that when $U_{t}$ increases from 5 to 7, the OP is reduced even for high values of *e*. This shows that the proposed scheduling scheme, in addition to improving the ZF detector performance, also reduces the undesirable effects of imperfect channel estimation in exchange for a slight increase in computational complexity.

**Figure 4:**
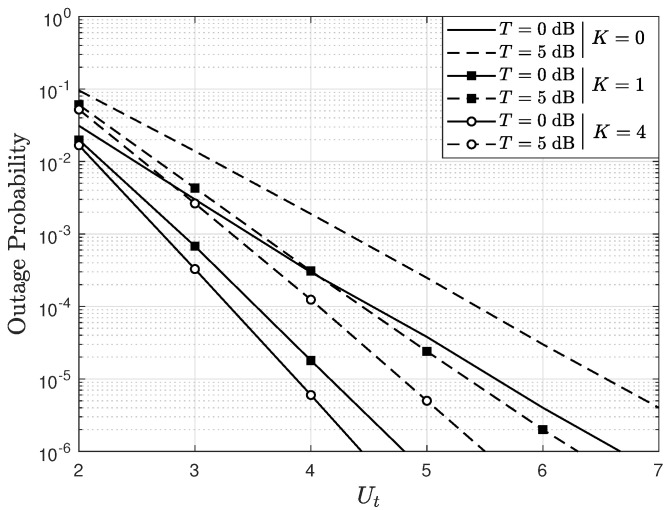
OP as a function of $U_{t}$, parameterized by *K* and the threshold *T*, considering BPSK modulation, A=2, U=2, and e=0.1.

Figure 4 shows the OP as a function of $U_{t}$, parameterized by *K* and *T* considering U=2, BPSK modulation, A=2, $E_{b}/N_{0}=15$ dB, and e=0.1. The results show that as $U_{t}$ increases, the OP decreases. Thus, it is evident that the proposed scheduling scheme improves the MU-MIMO system performance even though it is fully loaded, that is, when A=U. On the other hand, observe that the system performance improves when *K* increases. This is because the power of the LOS component between the transmitter and the receiver increases, which implies a channel with better propagation conditions. In particular, when K=0, the Rician fading becomes a Rayleigh fading channel. In this scenario, there are no line-of-sight components between the UTs and the BS, and it is the worst operation scenario, and therefore, the highest BER appears. Finally, when *T* increases, it is observed that the system performance worsens, since a higher value of *T* implies that a higher SNR is required for the system to work properly. Hence, if the SNR is not increased, then the OP increases.

### 5.2. Bit Error Rate

**Figure 5:**
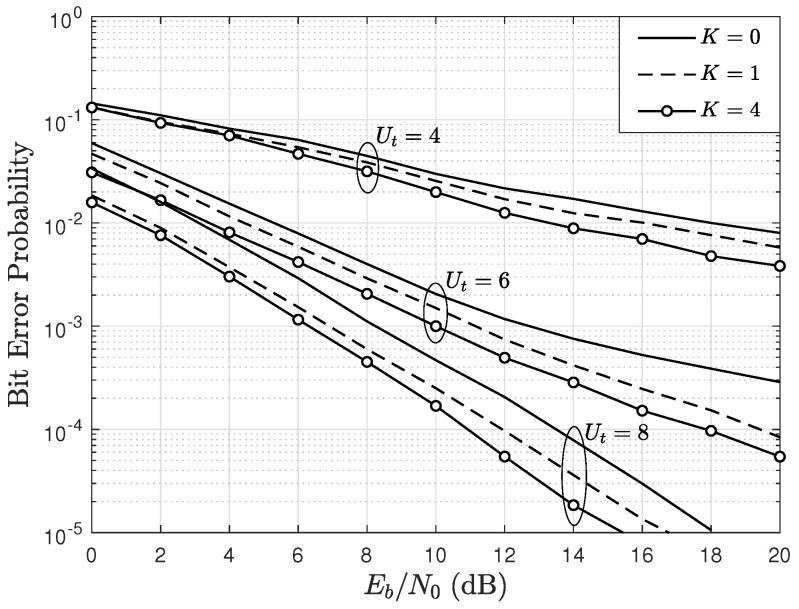
BER as a function of $E_{b}/N_{0}$, parameterized by $U_{t}$, and by the Rician factor *K*, considering BPSK modulation, A=4, U=4, and e=0.1.

Figure 5 shows the mean BER as a function of $E_{b}/N_{0}$, parameterized by *K* and $U_{t}$ considering A=4, U=4, BPSK modulation, e=0.1. The results show that, as the $E_{b}/N_{0}$ increases, the mean BER decreases. However, it is observed that the curves do not decay as quickly or linearly as is usually observed in scenarios with perfect channel estimation [8]. Thus, it is observed that the mean BER decays slowly as the $E_{b}/N_{0}$ increases. This behavior occurs due to the imperfect channel estimation at the BS receiver. Nevertheless, as *K* increases, the BER decreases, since the power of the LOS component of the signals increases. Additionally, for a given value of $E_{b}/N_{0}$, it is observed that when $U_{t}$ increases, then the mean BER decreases. Specifically, when $U_{t}=4$, the system is an ordinary MU-MIMO system in which the scheduling cannot be applied. On the other hand, when $U_{t}=6$, the proposed scheme is applied, and therefore performance improves. Additionally, when $U_{t}$ increases, it is observed that the inclination (slope) of the BER curves changes. Specifically, they decay faster as the $E_{b}/N_{0}$ increases, which signifies an increase in diversity despite the imperfect channel estimation and despite the system being fully loaded.

**Figure 6:**
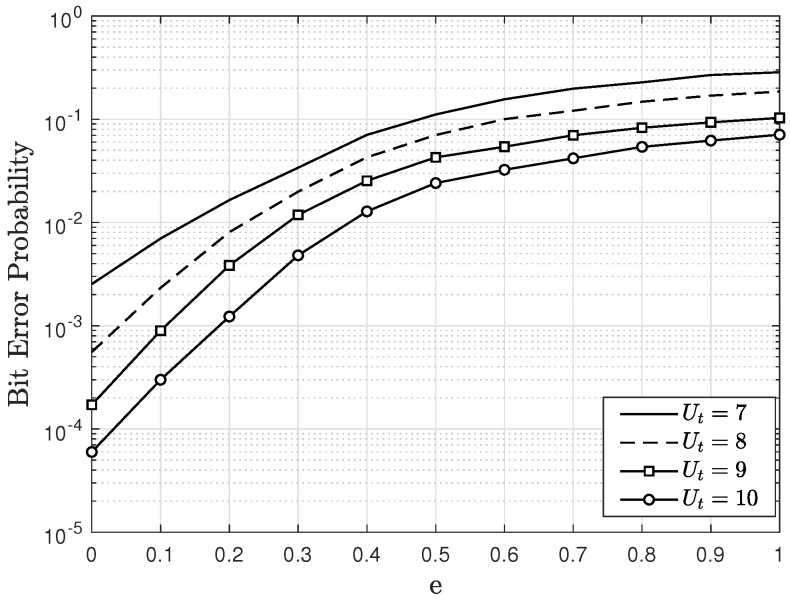
BER as a function of *e*, parameterized by $U_{t}$, considering BPSK modulation, A=6, U=6, $E_{b}/N_{0}=10$ dB and a channel with Rician factor K=2.

Figure 6 shows the mean BER as a function of *e*, parameterized by $U_{t}$ considering BPSK modulation, A=6, U=6, $E_{b}/N_{0}=10$ dB and a fading channel with a Rician factor K=2. The results show that as *e* approaches to one, the BER increases due to imperfections in the channel estimation. This event may cause some users to not be chosen properly in the receiver with the proposed scheduling scheme. However, when $U_{t}$ is increased, the system performance improves because the diversity also increases. These results corroborate that the proposed scheduling scheme also allows reducing the undesirable effects produced by the imperfect channel estimation. Thus, the higher $U_{t}$, the lower the BER of the system. Obviously, in exchange for an increase in the number of complex operations carried out by the receiver at the BS, but which can be performed in practical systems.

### 5.3. Comparison of the Proposed Scheduling Scheme with Other Schemes from Previous Works

Now, the presented proposal is compared with the scheduling schemes described in Section 4.1, that is the Frobenius norm-based scheduling algorithm and the fair channel allocation algorithm in terms of the mean BER. In particular, the Frobenius norm-based algorithm is known to be one of the suboptimal allocation schemes with best trade-off between implementation complexity and performance [27], for this reason, it is considered for comparative purposes. Moreover, the fair algorithm seeks an equitable transmission of all users in the MIMO system, as a consequence, it is also an interesting scheme to be compared with the algorithm proposed in this work.

**Figure 7:**
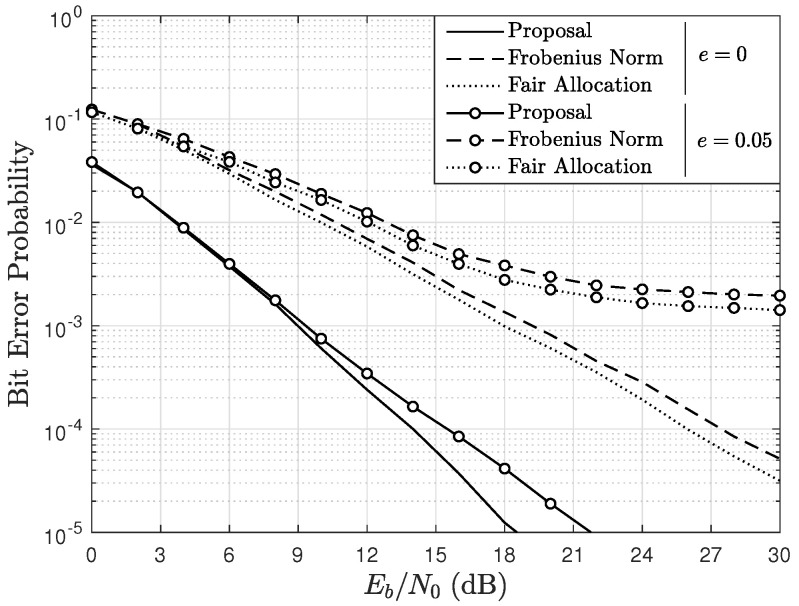
BER as a function of $E_{b}/N_{0}$, parameterized by *e*, and the scheduling algorithm considering BPSK modulation, A=4, U=4, $U_{t}=5$, and a channel with Rician factor K=2.

**Figure 8:**
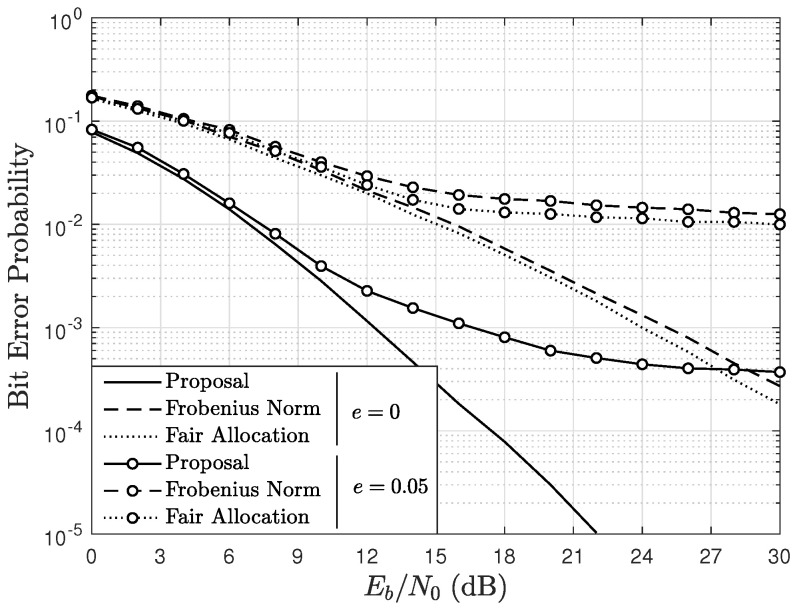
BER as a function of $E_{b}/N_{0}$, parameterized by *e*, and the scheduling algorithm considering 16-QAM, A=4, U=4, $U_{t}=5$, and a channel with Rician factor K=2.

Figure 7 and Figure 8 show the mean BER as a function of $E_{b}/N_{0}$, parameterized by *e*, and by the scheduling algorithm using A=4, U=4, and $U_{t}=5$ in a Rician fading channel with K=2. In particular, Figure 7 considers BPSK modulation and Figure 8 considers 16-QAM. In the figures, notice that both for e=0 and e=0.05, the BER obtained with the presented proposal is lower than that obtained with the Frobenius norm-based and fair allocation algorithms. This occurs because the proposed algorithm allows the system to obtain diversity, which is presented as a performance gain. On the other hand, the mean BER with the Frobenius norm-based algorithm is slightly lower than that obtained with the fair allocation algorithm. For e=0.05, the BER obtained with all the algorithms is considerably affected by the imperfect channel estimation, and this effect is greater when 16-QAM is used. However, for both BPSK and 16-QAM, the presented proposal obtains the lowest BER in this scenario. In addition, notice that the imperfect channel estimation generates floors in the BER curves which cannot be eliminated increasing the $E_{b}/N_{0}$. However, as $U_{t}$ is increased in the proposed scheduling scheme, the floors appear at lower BER values.

In the scenarios of Figure 7 and Figure 8, the number of complex operations performed by each algorithm does not change, since they are independent of the modulation order. Thus, from (20), the number of complex operations performed by the proposed algorithm is 750, from (15), the Frobenius norm-based algorithm carries out 320 complex operations and, from (16), the fair allocation algorithm performs 80 complex operations. Thus, although the proposed algorithm is the one that performs a greater number of operations, it is also interesting to observe that, for both modulation schemes, the BER obtained with the presented proposal in the imperfect channel estimation scenario with e=0.05 is lower than the BER obtained with the other algorithms operating in a perfect channel estimation scenario (e=0) for certain values of $E_{b}/N_{0}$.

### 5.4. Secrecy Outage Probability

Finally, in this subsection, it is verified how the proposed scheme can help reduce the SOP in case of presence of a spy external receiver (eavesdropper) that tries to decode the information sent by the UTs in the MU-MIMO system. Thus, the analysis performed considers $U_{t}$ users who try to connect to a trusted BS, of which only *U* connect to it according to the proposed scheduling algorithm. In addition, there is also an eavesdropper BS that tries to decode the information of the *U* users in the MIMO system.

In particular, the SOP is defined as the probability that the secrecy capacity is below a given threshold value [32,52], where the secrecy capacity is defined as the channel capacity obtained with the SNR of the trusted BS minus the channel capacity obtained with the untrusted receiver’s SNR. Thus, the lower the SOP, the greater the protection of the information.

From [53] and (11), the achievable rate of the BS decoding the signal of the *k*-th user can be obtained as

(21) CB,k≜log21+EbN01[(H^BHH^BH)−1]k,klog2M,

where ${\hat{H}}_{B}$ is an A×U matrix containing the estimated channels gains for the links between the UTs and the BS after the scheduling process. The entries of ${\hat{H}}_{B}$ have mean power *P*, given by (4). Similarly, the achievable rate of the eavesdropper decoding the signal of the *k*-th UT can be obtained as

(22) CE,k≜log21+EbN01[(H^EHH^EH)−1]k,klog2M,

where ${\hat{H}}_{E}$ is an $A_{E}\times U$ matrix containing the estimated channels gains for the links between the UTs and the eavesdropper. Thus, notice that the number of receiving antennas at the trusted BS and at the eavesdropper can be different. The entries of ${\hat{H}}_{E}$ have mean power $P_{E}=cP$, where 0<c≤1 modifies the mean power of the eavesdropper channel gains. Thus, a small value of *c* can be interpreted as the distance between the UTs and the eavesdropper being much larger than the distance between the UTs and the trusted BS. At the other extreme, c=1 implies that the BS and the eavesdropper are at the same distance from the UTs. Moreover, no scheduling process based on the elements of ${\hat{H}}_{E}$ is performed, and the elements of ${\hat{H}}_{E}$ are independent from the elements of ${\hat{H}}_{B}$. The non-negative secrecy capacity for the *k*-th UT can be obtained as

(23) $$C_{k}={\left[C_{B,k}-C_{E,k}\right]}^{+},$$

where ${\left[x\right]}^{+}=max\left(x,0\right)$. Then, the SOP for the *k*-th UT is given by

(24) $$SOP_{k}=P\left(C_{k}\leq R\right),$$

where *R* denotes the secrecy target rate to guarantee the security of the *k*-th UT in the MIMO system.

**Figure 9:**
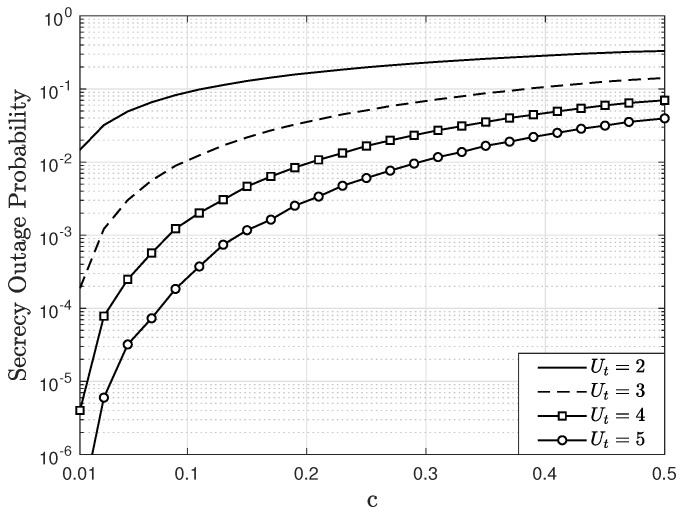
SOP as a function of *c*, parameterized by $U_{t}$, considering M=4, A=2, $A_{E}=2$, U=2, $E_{b}/N_{0}=15$ dB, e=0.1, and R=1 bps in a Rician fading channel with K=2.

**Figure 10:**
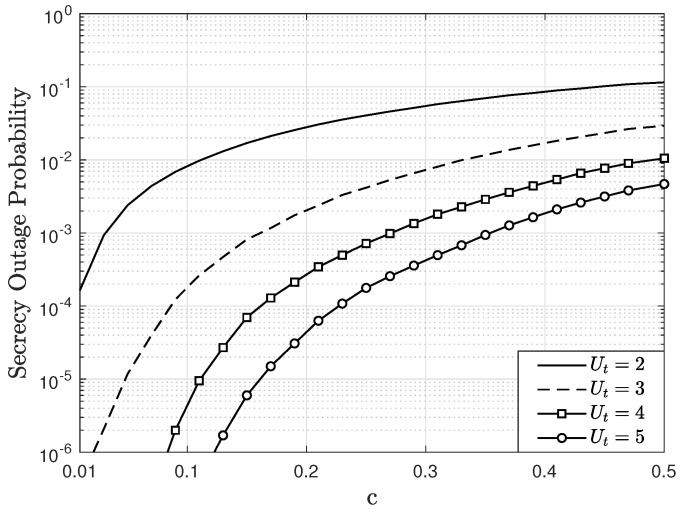
SOP as a function of *c*, parameterized by $U_{t}$, considering M=4, A=3, $A_{E}=2$, U=2, $E_{b}/N_{0}=15$ dB, e=0.1, and R=1 bps in a Rician fading channel with K=2.

By considering the above results, Figure 9 and Figure 10 show the SOP as a function of *c*, parameterized by $U_{t}$, considering M=4, $A_{E}=2$, U=2, $E_{b}/N_{0}=15$, and e=0.1 in a Rician fading channel with K=2. For the SOP calculation, R=1 bps is employed and, without loss of generality, it is considered that the UT k=1 for the analysis. Figure 9 considers A=2 antennas at the trusted BS and Figure 10 considers A=3 antennas at the trusted BS. In both figures, the SOP increases as *c* increases, since the mean power of the fading channel for the paths between the eavesdropper and the UTs increases and consequently, its achievable rate increases. In other words, the eavesdropper can decode the information sent by the users more easily. On the other hand, for small values of *c*, or equivalently, when the eavesdropper is away from the UTs, the SOP decreases. For all scenarios, it is observed that when $U_{t}$ increases, then the SOP decreases. Specifically, when $U_{t}$ increases, the proposed scheduling scheme allows maximization of the the SNR of the users selected for transmitting. Therefore, the UT’s achievable rate, given by (21), increases, which implies a lower SOP. When comparing Figure 9 and Figure 10, the lowest SOP values appear in the last figure. For instance, for c=0.2 and $U_{t}=5$, the SOP in Figure 9 is equal to $3\times 10^{-3}$ and in Figure 10 is equal to $4.1\times 10^{-5}$. This occurs because the trusted BS employs one more antenna than the eavesdropper’s receiver. Despite this, it is observed that when $U_{t}$ increases, the SOP still decreases. Therefore, these results show that the proposed scheduling algorithm, together with other techniques, allow for an improved PLS of MIMO systems. It is important to indicate that if the eavesdropper is not aware of the channel state information between the UTs and the BS, he cannot know which users are transmitting, because the user selection is made based on this information. Thus, this ensures an additional degree of freedom to protect vulnerable information transmitted by certain users in the MIMO system.

**Figure 11:**
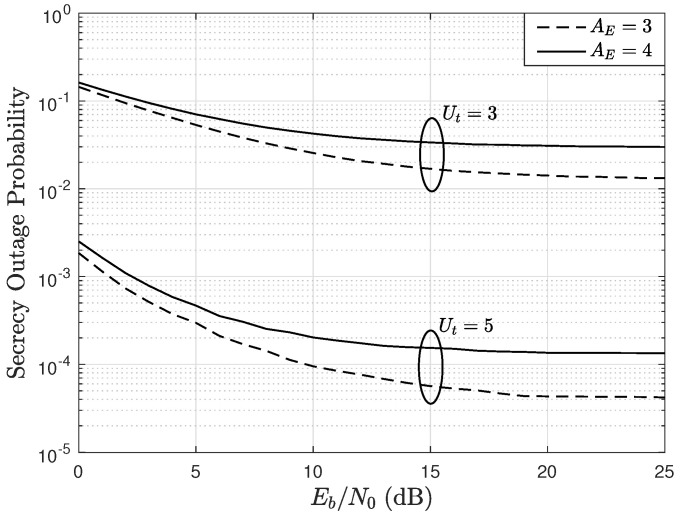
SOP as a function of $E_{b}/N_{0}$, parameterized by $A_{E}$ and $U_{t}$, considering M=4, A=3, U=3, e=0.1, c=0.05, and R=1 bps in a Rician fading channel with K=2.

Finally, Figure 11 shows the impact of the number of receiving antennas at the eavesdropper receiver, i.e., $A_{E}$, on the secrecy performance of the MIMO system. Thus, this figure shows the OP as a function of the $E_{b}/N_{0}$, parameterized by $A_{E}$ and $U_{t}$, considering M=4, A=3, U=3, e=0.1, c=0.05, and R=1 bps in a Rician fading channel with K=2. In the results, notice that as $A_{E}$ increases, the OP also increases. This occurs because the eavesdropper has a greater capacity to decode the information of the users, and this is due to the fact that its diversity increases. In addition, it is observed that there appears a floor in the the OP curves that cannot be reduced as the $E_{b}/N_{0}$ increases. In fact, an increase in the $E_{b}/N_{0}$ is associated with an increase in transmission power of the user terminals, which benefits both the trusted BS and the eavesdropper, resulting in no change in the OP curves. However, notice that as the $U_{t}$ is increased from 3 to 5, the OP is dramatically reduced. In particular, when $U_{t}=3$, a scheduling process is not carried out since $U_{t}=U$. On the other hand, when $U_{t}=5$, the scheduling is performed and, as a consequence, the MIMO system security is improved.

## 6. Conclusions

In this work, a scheduling scheme for the uplink of MU-MIMO systems using *A* antennas and the ZF multi-user detector in the receiver was proposed and evaluated over a Rician fading channel in the presence of imperfect channel estimation. With the proposed scheme, the system selects *U* users from a group of $U_{t}>U$ users, who transmit simultaneously on the same radio resource so that the SNR is maximized. The computational complexity of the proposed scheme was determined in terms of the number of complex operations, and its performance was evaluated in terms of the OP and the BER.

It was determined that as $U_{t}$ increases, the OP and the BER decrease, which implies that the proposed scheme improves the performance of MU-MIMO systems. Furthermore, it was observed that the system diversity increases when $U_{t}$ increases. Thus, the proposed scheme along with the ZF detector eliminates interference and increases system diversity even when it is fully loaded, that is, when A=U. The results also show that the proposed scheme mitigates the undesirable effects produced by channel estimation errors, and this mitigation is greater as $U_{t}$ increases. Finally, since the proposed scheme allows maximization of the SNR of the users, it also allows an increase in their achievable rate, which in a scenario in the presence of an eavesdropper, allows a reduction of the SOP of the MU-MIMO system.

In this work, the ZF detector was considered due to its low computational complexity and good performance. However, there are other multiuser detectors on which the proposed scheme can also be applied. Therefore, this is an option for future work. In addition, there are other more general distributions to model the fading phenomenon, such as α−μ or κ−μ distributions, that are adequate for propagation environments in millimeter wave scenarios [54]. Thus, the analysis of the proposed scheme in these channels is also an alternative for future research.

## Figures and Tables

**Table 1:** Related work summary.

MIMO Research Area	Previous Works									[P]
	[7,10]	[8]	[14]	[16,17]	[19]	[20,21,22,23,24,25,26]	[27]	[31]	[32,33,34]	
Performance evaluation in Rayleigh fading	X	X	X	X	X	X	X	X	X	X
Performance evaluation in generalized fading			X	X		X		X	X	X
Imperfect channel estimation	X	X		X						X
Multiuser detection		X			X					X
Scheduling algorithms						X	X		X	X
Physical layer security								X	X	X

[P] = Proposal presented in this work.

## Data Availability

Not applicable.
